# Involvement of SIRT3‐GSK3β deacetylation pathway in the effects of maternal diabetes on oocyte meiosis

**DOI:** 10.1111/cpr.12940

**Published:** 2020-10-26

**Authors:** Yongan Xin, Yifei Jin, Juan Ge, Zhenyue Huang, Longsen Han, Congyang Li, Danni Wang, Shuai Zhu, Qiang Wang

**Affiliations:** ^1^ State Key Laboratory of Reproductive Medicine Suzhou Municipal Hospital Nanjing Medical University Nanjing China; ^2^ School of Nursing Nanjing Medical University Nanjing China; ^3^ Center for Global Health School of Public Health Nanjing Medical University Nanjing China

**Keywords:** diabetes, meiosis, oocyte, oxidative stress, Sirtuin

## Abstract

**Objectives:**

It has been widely reported that maternal diabetes impairs oocyte quality. However, the responsible mechanisms remain to be explored. In the present study, we focused on whether SIRT3‐GSK3β pathway mediates the meiotic defects in oocytes from diabetic mice.

**Materials and methods:**

GSK3β functions in mouse oocyte meiosis were first detected by targeted siRNA knockdown. Spindle assembly and chromosome alignment were visualized by immunostaining and analysed under the confocal microscope. PCR‐based site mutation of specific GSK3β lysine residues was used to confirm which lysine residues function in oocyte meiosis. siRNA knockdown coupled with cRNA overexpression was performed to detect SIRT3‐GSK3β pathway functions in oocyte meiosis. Immunofluorescence was performed to detect ROS levels. T1DM mouse models were induced by a single intraperitoneal injection of streptozotocin.

**Results:**

In the present study, we found that specific depletion of GSK3β disrupts maturational progression and meiotic apparatus in mouse oocytes. By constructing site‐specific mutants, we further revealed that acetylation state of lysine (K) 15 on GSK3β is essential for spindle assembly and chromosome alignment during oocyte meiosis. Moreover, non–acetylation‐mimetic mutant GSK3β‐K15R is capable of partly preventing the spindle/chromosome anomalies in oocytes with SIRT3 knockdown. A significant reduction in SIRT3 protein was detected in oocytes from diabetic mice. Of note, forced expression of GSK3β‐K15R ameliorates maternal diabetes‐associated meiotic defects in mouse oocytes, with no evident effects on oxidative stress.

**Conclusion:**

Our data identify GSK3β as a cytoskeletal regulator that is required for the assembly of meiotic apparatus, and discover a beneficial effect of SIRT3‐dependent GSK3β deacetylation on oocyte quality from diabetic mice.

## Graphical abstract

Diabetic oocytes display a reduction in SIRT3 protein and show higher frequency of spindle/chromosome defects and excessive ROS. Deacetylation‐mimetic mutant GSK3β‐K15R alleviates the meiotic defects in oocytes from diabetic mice, while it was unable to decrease the ROS level in diabetic oocytes.

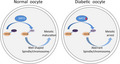

## 1 INTRODUCTION

Type 1 diabetes mellitus is a global health issue, and its pooled prevalence has already reached to 2.69‰ and increased at the rate of 1.8% annually.^1^, ^2^ Animal models show that diabetes induces abnormal redistribution of endoplasmic reticulum,^3^ mitochondrial dysfunction, meiotic apparatus disorganization,^4^, ^5^ reduced thickness of zona pellucida^6^ and epigenetic changes in oocytes.^6^, ^7^, ^8^, ^9^ In addition, diabetes condition leads to defective energy metabolism in oocyte^5^, ^10^, ^11^ and preimplantation embryo.^12^ Within cumulus cells, inappropriate apoptosis and mitochondrial dysfunction are also observed in diabetes mouse.^13^ These factors contribute to the impaired oocyte quality, consequently influencing embryo development and pregnancy outcomes. Although the defective phenotypes have been identified in oocytes from diabetic animals, the underlying molecular pathways have yet to be determined.

Sirtuins (SIRT1‐7), a family of NAD^+^‐dependent deacetylases, are involved in multiple biological processes including energy metabolism, oxidative stress, cellular ageing and longevity.^14^, ^15^, ^16^, ^17^, ^18^, ^19^ Sirtuins are increasingly identified as key mediators in the control of gametogenesis, fertilization and embryo development since a sterile phenotype was observed in SIR2α (silent information regulator 2) null mice.^15^, ^20^ Among Sirtuin family, SIRT3 is located in mitochondrial matrix^21^ and maintains metabolism homeostasis under basal or stress condition through deacetylating various enzymes, such as AcsCS2, LCAD, OTC and HMGCS2.^22^, ^23^ In addition, SIRT3 has been shown to be able to control the antioxidant system via regulating superoxide dismutase 2 (SOD2) during mammalian oocyte maturation.^24^, ^25^, ^26^, ^27^ Recently, Nagalingam et al^28^ revealed that SIRT3 could deacetylate glycogen synthase kinase‐3 beta (GSK3β), enhancing its enzymatic activity. GSK3β is a highly conserved serine/threonine protein kinase and linked to several cellular events and some prevalent diseases.^29^, ^30^ Genetic mutation study demonstrated that loss of function of GSK3β in *Drosophila* neuroblasts causes mitotic apparatus anomalies and delayed metaphase‐to‐anaphase transition.^31^ Baluch et al^32^ found that GSK3β is accumulated at both spindle poles and kinetochore region in metaphase oocytes. In addition, inhibition of GSK3β leads to massive apoptosis of meiotic prophase I oocyte via premature TAp63 expression.^33^ However, to date, little is known about the function of GSK3β and its interaction with SIRT3 during oocyte maturation.

In the present study, by knockdown and overexpression experiments, we uncovered the role of GSK3β in oocyte meiosis and showed that SIRT3‐GSK3β deacetylation pathway functions in the defective phenotypes of oocytes from diabetic mice.

## 2 MATERIALS AND METHODS

All chemicals and culture media were purchased from Sigma unless stated otherwise.

### 2.1 Mice and ethics statement

Female ICR mice (3‐4 weeks old) were sacrificed in this study. Mouse models of T1DM were induced by a single intraperitoneal injection of streptozotocin (S0130; Sigma‐Aldrich) at a dose of 190 mg/kg after fasting 12 hours. Four days later, blood glucose was measured via a glucometer. Mice with glucose level higher than 300 mg/dL (16.7 mmol/L) were selected for further analysis. Controls were injected with an equivalent volume of sodium‐citrate solution to account for the solvent (S4641; Sigma‐Aldrich). All procedures and animal care followed guidelines stipulated by the Care and Use Committee of Nanjing Medical University.

### 2.2 Antibodies

The following antibodies were used in this study: rabbit polyclonal anti‐SIRT3 (Cat#:ab8667; Abcam); rabbit monoclonal anti‐GSK3β (Cat#:ab32391; Abcam); mouse monoclonal FITC‐conjugated anti‐α‐tubulin antibody (Cat#: F2168; Sigma); rabbit monoclonal anti‐Myc antibody (Cat#: 2278; Cell Signaling Technology); mouse monoclonal HRP‐conjugated anti‐α‐tubulin antibody (Cat#: HRP‐66031; Proteintech).

### 2.3 Oocyte collection and culture

Female mice were superovulated by intraperitoneal injection with 5 IU pregnant mares' serum gonadotrophin (PMSG) ~48 hours prior to oocyte collection. Cumulus‐oocytes complexes (COCs) were dissociated by repeatedly puncturing of the ovary surface using syringe needle and collected using mouth‐controlled micropipette. Cumulus cells surrounding oocyte were separated by pipetting the complex up and down using micropipette with inner diameter of ~100 μm. For in vitro *maturation*, GV oocytes were cultured in paraffin oil covered M16 medium in incubator (37°C, 5% CO_2_, 5% O_2_, 90% N_2_).

### 2.4 Immunofluorescence

Visualization of spindle and chromosomes was conducted as described previously.^34^ In brief, oocytes were fixed in 4% fresh‐prepared paraformaldehyde for 30 minutes and permeabilized in 0.5% Triton X‐100 for 20 minutes at room temperature. Following incubation in blocking buffer (PBS, 0.5% Triton X‐100, 1% BSA) for 1 hour, oocytes were labelled with FITC‐conjugated anti‐tubulin antibody overnight at 4°C. After three washes, oocytes were stained with propidium iodide (PI) for 20 minutes to visualize chromosome. Finally, oocytes were transferred to microscope slides and examined under a laser scanning confocal microscope in time (LSM 700, Zeiss).

### 2.5 Measurement of intracellular ROS

Intracellular ROS level was detected by CM‐H_2_DCFDA probe (Cat#: C6827; Life Technologies). Oocytes were incubated in HEPES buffer containing 5 µmol/L CM‐H_2_DCFDA for 30 minutes. After three washes with fresh HEPES buffer, 10 oocytes were loaded on Nunc™ Glass Bottom Dishes (Cat#:15082; Thermo Scientific) and covered with mineral oil. The images are acquired using a laser scanning confocal microscope (LSM 700, Zeiss).

### 2.6 Plasmid construction and mRNA synthesis

Total RNA from oocyte sample was extracted and reverse‐transcribed into cDNA as we described previously.^35^ PCR products were purified and digested with FseI and AscI (NEB Inc), and then inserted into the pCS2 + plasmid vector encoding N‐terminal Myc‐tags. GSK3β mutant plasmids (K15Q, K15Q, K36Q and K36R) were generated through PCR using Phusion High‐Fidelity DNA Polymerase (Cat#: M0530S, NEB). For mRNA synthesis, the plasmids were linearized by Not I and capped RNAs were produced using SP6 mMESSAGE mMACHINE (Ambion) according to the manufacturer's instruction. Synthesized RNA was purified by Arcturus PicoPure RNA Isolation Kit (Applied Biosystems) and stored at −80°C. The related primers can be found in Table S1.

### 2.7 Knockdown or overexpression experiments

Microinjection experiments were conducted using a Narishige microinjector for SIRT3 knockdown or overexpression of GSK3β mutants. siRNA duplexes against SIRT3 and GSK3β were purchased from Gene Pharma and diluted to 20 µmol/L stock solutions. Five picolitre solution of siRNA or capped RNA was injected into the cytoplasm of oocytes. Control oocytes were injected with the same amount of negative control or PBS in parallel. After injections, oocytes were cultured in M16 medium supplemented with 2.5 µmol/L milrinone for 20‐22 hours to make oocytes arrest at GV stage, facilitating the degradation or translation of targeted RNA, and then moved to milrinone‐free medium for further analysis. The related siRNA sequences are listed in Table S1.

### 2.8 Western blot

The samples containing sufficient number of oocytes (at least 100) are lysed in Laemmli buffer and denatured by boiling for 5 minutes before electrophoresis. The denatured proteins were then separated by 12% precast SDS‐PAGE gel and electrically transferred to polyvinylidene fluoride (PVDF) membranes. After blocking with 5% skimmed milk for 1 hour, membranes were incubated with primary antibodies overnight at 4°C (anti‐SIRT3 pAb, 1:500; anti‐GSK3β mAb, 1:1000; anti‐Myc‐Tag mAb, 1:1000), followed by three washes with PBST (PBS containing 0.1% Tween 20) and incubation with HRP‐conjugated secondary antibody for 1 hour at room temperature. The protein bands were visualized by an ECL Plus Western Blotting Detection System (GE Healthcare). For loading control, membranes were rinsed in stripping buffer and re‐probed with anti‐α‐tubulin antibody (1:2000).

### 2.9 Quantitative real‐time PCR

Quantitative real‐time PCR (qRT‐PCR) was performed to verify the knockdown efficiency of siRNA. In brief, total RNA was isolated from control or knockdown oocytes using an Arcturus PicoPure RNA Isolation Kit (Applied Biosystems), and reverse‐transcribed with PrimeScript RT Master Mix (TaKaRa). The cDNA was quantified by SYBR Green Mix (Vazyme) using StepOnePlus™ Real‐Time PCR System (Applied Biosystems). GAPDH was used as an internal control. Primer sequences are listed in Table S1.

### 2.10 Statistical analysis

GraphPad Prism software was used to analyse data. Data are presented as mean ± SD, unless otherwise indicated. Differences were analysed by Student's *t* test. *P* values <.05 were considered to be significant. At least three replicates were conducted for each treatment.

## 3 RESULTS

### 3.1 GSK3β is required for mouse oocyte meiosis

**FIGURE 1:**
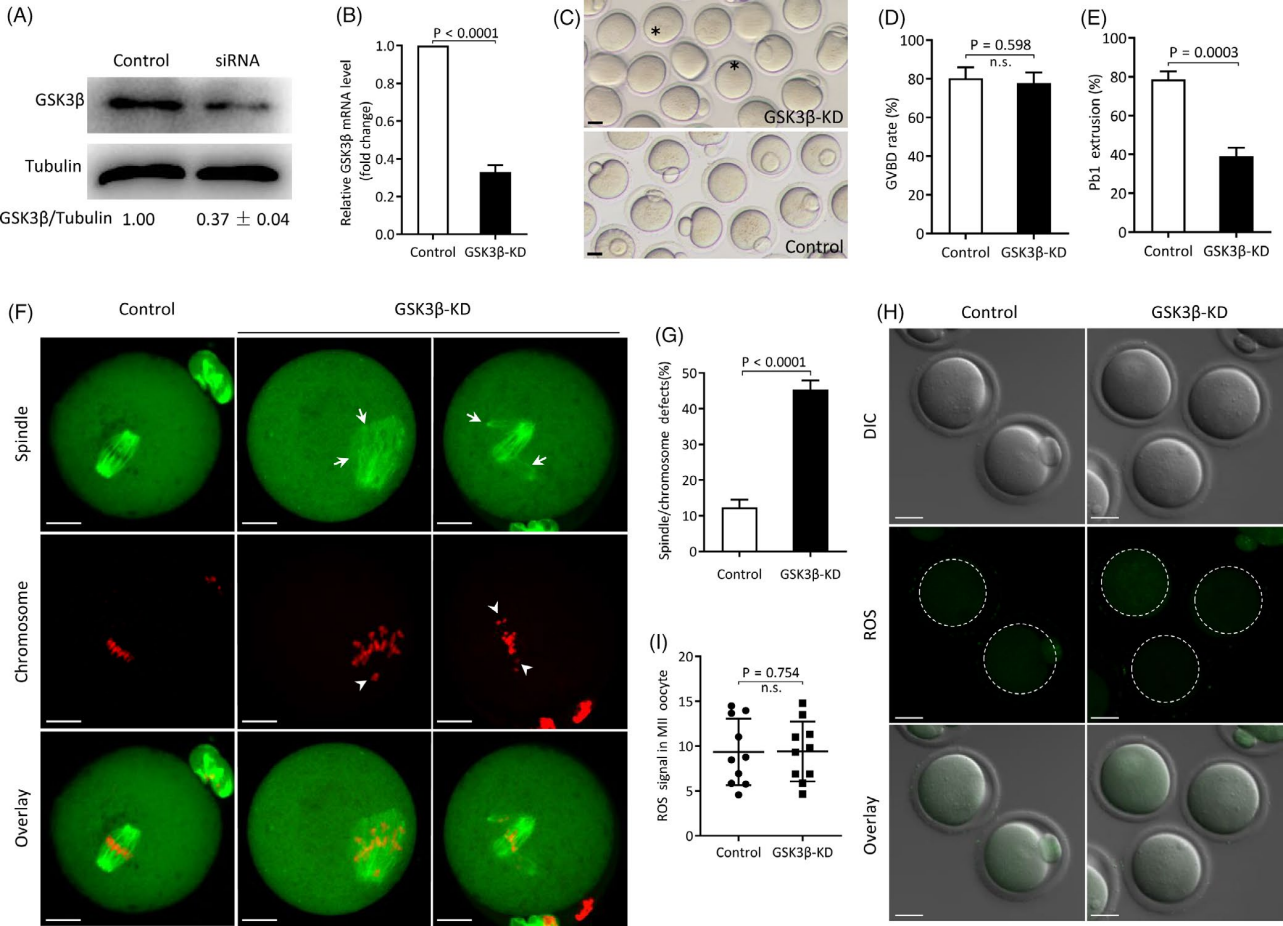
Effects of GSK3β knockdown on oocyte maturation. A, B, Knockdown efficiency of GSK3β‐siRNA was verified by Western blot and qRT‐PCR, respectively. Tubulin or GAPDH served as an internal control. Western blot experiments were repeated at least three times, with a representative gel image shown. C, Phase‐contrast images of control and GSK3β‐KD oocytes. Asterisks indicate the oocytes that fail to extrude polar body, Scale bars: 30 μm. D, E, Rate of GVBD and Pb1 extrusion in control and GSK3β‐KD oocytes. F, Representative confocal images of control and GSK3β‐KD oocytes stained with α‐tubulin antibody (green) and propidium iodide (red) to visualize spindle and chromosome, respectively. Oocyte with typical bipolar spindle and well‐aligned chromosome is regarded as normal. Oocytes showing spindle disorganization (arrows) and chromosome misalignment (arrowheads) are easily observed in GSK3β‐KD oocytes. Scale bars: 20 μm. G, Percentage of oocytes with abnormal spindle/chromosomes in control and GSK3β‐KD group. Data are expressed as mean ± SD from three independent experiments in which at least 100 oocytes were examined. H, ROS signal was detected by CM‐H_2_DCFDA fluorescence dye and captured using laser confocal microscope. Representative images of ROS signal (green) in control and GSK3β‐KD oocytes. Scale bars: 30 μm. I, Quantification of ROS fluorescence intensity (n = 10 for each group)

Accumulation of GSK3β at both spindle poles and kinetochore region has been reported in mouse oocytes.^32^ To explore the role of GSK3β during oocyte maturation, we microinjected GSK3β siRNA into fully grown mouse oocytes. This led to a significant knockdown (KD) of both GSK3β protein and mRNA (Figure 1A,B). Although oocytes from control and GSK3β‐KD group had comparable germinal vesicle breakdown (GVBD) rate (77.7 ± 5.7% vs 80.3 ± 5.6% control; *P* > .05; Figure 1D), markedly reduced polar body (Pb1) extrusion rate was detected in GSK3β‐KD oocytes (39.0 ± 4.5% vs 78.7 ± 4.0% control; *P* < .05; Figure 1C,E). Spindle assembly is important for correct chromosome segregation and asymmetric division in oocytes.^36^, ^37^ Given that most oocytes fail to complete meiosis I and extrude Pb1 in the GSK3β‐KD group, we speculated that the assembly of meiotic apparatus is disturbed. To this end, oocytes were immunolabelled with anti‐tubulin antibody to visualize the spindle and co‐stained with propidium iodide for chromosomes. As shown in Figure 1F,G, the percentage of oocytes exhibiting disorganized spindle with randomly scattered chromosomes in the GSK3β‐KD group was considerably higher than control oocytes showing chromosomes tightly align at the equator plane of barrel‐shaped spindle (45.4 ± 2.6% vs 12.3 ± 2.2% control; *P* < .05). Oxidative stress has been suggested to induce microtubule instability.^38^ Hence, we decided to assess reactive oxygen species (ROS) level in GSK3β‐KD oocytes using CM‐H_2_DCFDA fluorescence dye. Nevertheless, as shown in Figure 1H,I, both control and GSK3β‐KD oocytes displayed negligible fluorescence signal (9.40 ± 3.34 vs 9.35 ± 3.71 control; *P* > .05), indicating that GSK3β depletion has no effects on redox homeostasis in mouse oocytes. Altogether, the results suggest that proper spindle assembly and chromosome alignment in oocytes requires GSK3β.

### 3.2 Acetylation‐mimetic mutant GSK3β‐K15Q disrupts meiotic apparatus in mouse oocytes

**FIGURE 2:**
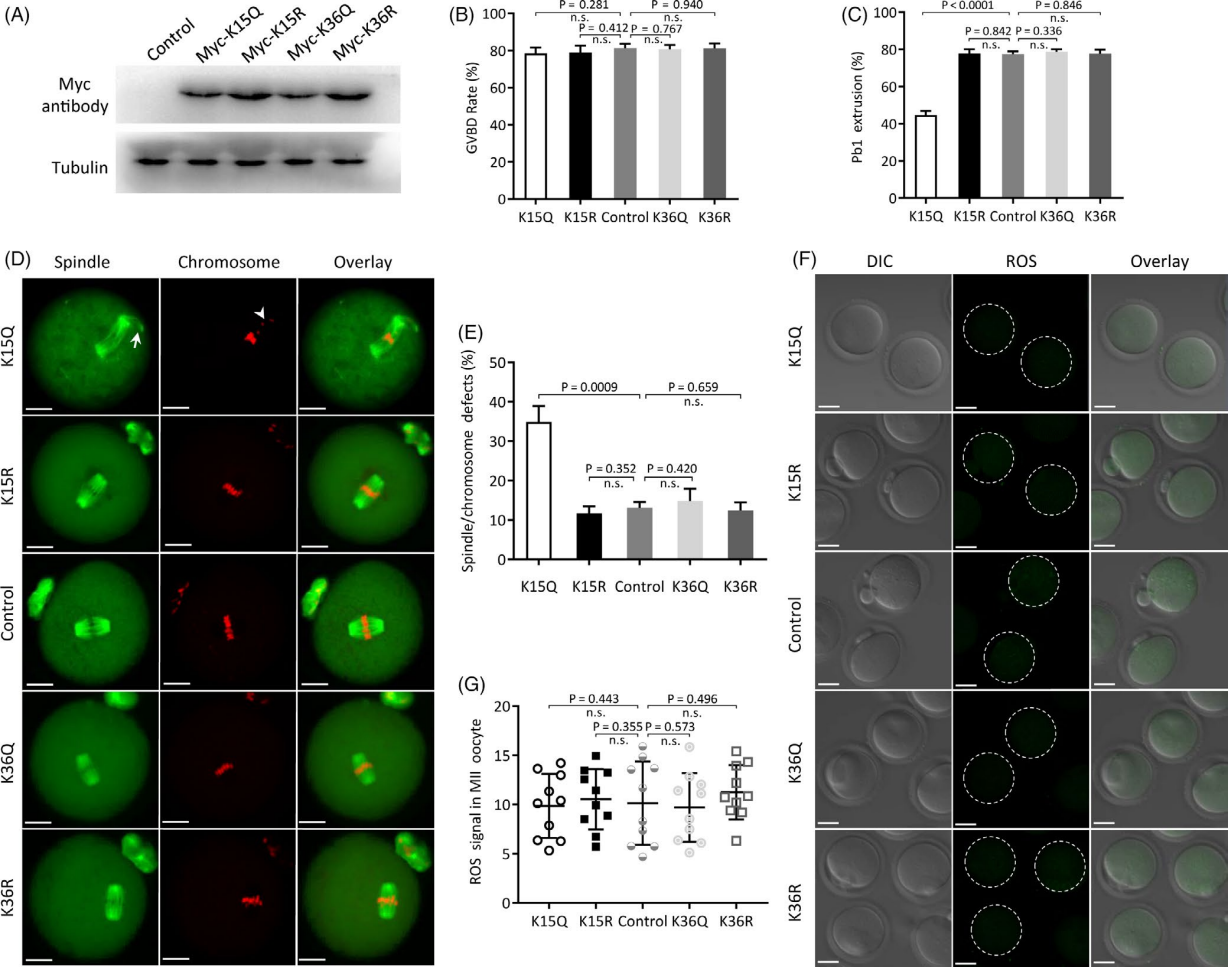
Effects of GSK3β acetylation on oocyte maturation. Acetylation‐mimetic mutant GSK3β‐K15Q/K36Q or deacetylation‐mimetic mutant GSK3β‐K15R/K36R was microinjected into fully grown oocytes to evaluate meiotic apparatus and redox homeostasis. A, Western blotting shows that four mutants of GSK3β were efficiently overexpressed, probing with anti‐Myc antibody. B, C, Quantitative analysis of GVBD (control: 81.3 ± 2.3%, K15Q: 78.6 ± 3.1%, K15R: 79.0 ± 3.7%, K36Q: 80.7 ± 2.3%, K36R: 81.2 ± 2.7%)and Pb1 extrusion rate (control: 77.5 ± 1.5%, K15Q: 44.7 ± 2.1%, K15R: 77.8 ± 2.3%, K36Q: 78.8 ± 1.3%, K36R: 77.8 ± 2.0%) in control and GSK3β mutant oocytes. D, Representative images of spindle/chromosomes in control and GSK3β mutant oocytes. Arrows indicate spindle defects, and arrowheads indicate chromosome misalignment. Scale bars: 20 μm. E, Percentage of oocytes with abnormal spindle/chromosomes in control and GSK3β mutant groups (control: 13.1 ± 1.5%, K15Q: 34.9 ± 4.0%, K15R: 11.7 ± 1.8%, K36Q: 14.9 ± 3.1%, K36R: 12.4 ± 2.1%). Data are expressed as mean ± SD from three independent experiments in which at least 100 oocytes were examined. F, Representative images of ROS signal (green) in control and GSK3β mutant oocytes. Scale bars: 30 μm. G, Quantification of fluorescence intensity (control: 10.14 ± 4.24, K15Q: 9.85 ± 3.25, K15R: 10.53 ± 3.10, K36Q: 9.69 ± 3.49, K36R: 11.25 ± 2.76; n = 10 for each group)

Recent work indicated that GSK3β activity is regulated by reversible acetylation at specific lysine (K) reside.^28^ To investigate whether GSK3β acetylation, and if so, which lysine residues function in oocyte meiosis, site‐specific mutants targeting K15 and K36 were constructed and microinjected into fully grown oocytes for phenotypic analysis. Substitution of lysine (K) with a glutamine (Q) mimics an acetylated amino acid state, while substitution with an arginine (R) mimics deacetylation.^39^ As shown in Figure 2A, GSK3β mutants (K15Q, K15R, K36Q and K36R) were ectopically expressed in oocytes, confirmed by Western blotting. For brevity, these oocytes are called ‘K15Q/R oocytes’ and ‘K36Q/R oocytes’ here. We found that control and all mutant oocytes resume meiosis normally after 3 hours culture (Figure 2B); however, more than half of K15Q oocytes fail to exclude Pb1 following 14 hours culture (44.7 ± 2.1% vs 77.5 ± 1.5% control; *P* < .05; Figure 2C). In line with this, K15Q oocytes displayed higher frequency of spindle defects and chromosome misalignment than controls (34.9 ± 4.0% vs 13.1 ± 1.5% control; *P* < .05; Figure 2D,E). In contrast, overexpression of all mutants had little effects on ROS generation in oocytes (Figure 2F,G). Collectively, these data strongly suggest that acetylation status of K15 is important for GSK3β function in oocyte meiosis.

### 3.3 SIRT3 promotes the assembly of meiotic apparatus in mouse oocytes through deacetylation of GSK3β‐K15

**FIGURE 3:**
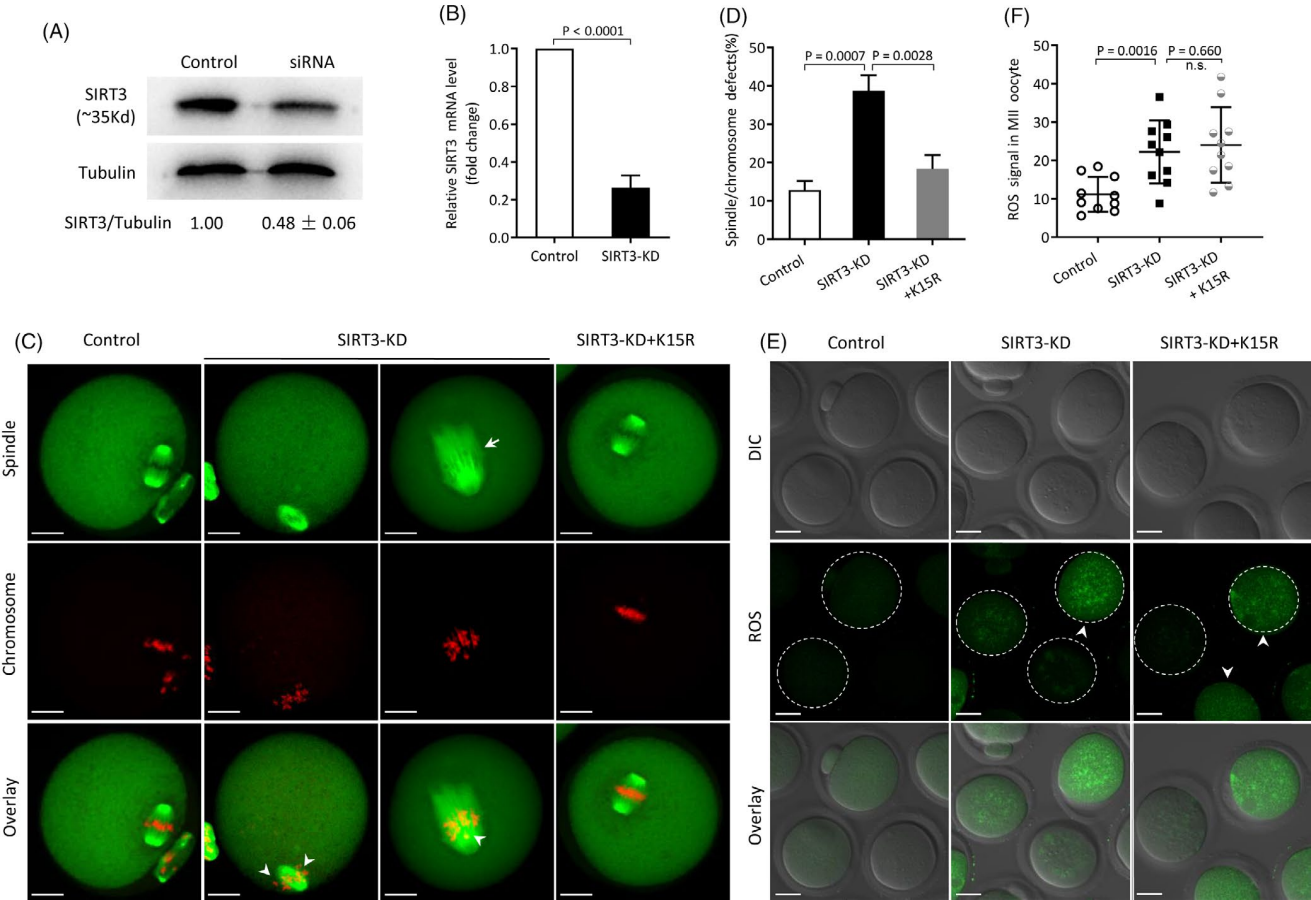
GSK3β‐K15R partly rescues the meiotic defects in SIRT3‐KD oocytes. A, B, Efficiency of SIRT3 knockdown was verified by Western blot and qRT‐PCR, respectively. Western blot experiments were repeated at least three times, with a representative gel image shown. C, Representative images of spindle/chromosomes in control, SIRT3‐KD and SIRT3‐KD + K15R oocytes, respectively. Arrows indicate spindle defects, and arrowheads indicate chromosome misalignment. Scale bars: 20 μm. D, Percentage of oocyte with abnormal spindle/chromosomes in control, SIRT3‐KD and SIRT3‐KD + K15R group. Data are expressed as mean ± SD from three independent experiments in which at least 100 oocytes were examined. E, Representative images of ROS signal (green) in control, SIRT3‐KD and SIRT3‐KD + K15R oocytes. Scale bars: 30 μm. Arrowhead indicates the significantly elevated fluorescence intensity. F, Quantification of ROS fluorescence intensity (n = 10 for each group)

SIRT3 was shown to be able to deacetylate GSK3β and thereupon upregulate its catalytic activity.^28^ Previously, we observed the meiotic defects and oxidative stress in oocytes depleted of SIRT3.^24^ Given the disorganized spindle/chromosomes in GSK3β‐K15Q oocytes, we speculated that SIRT3‐GSK3β deacetylation pathway may be involved in oocyte meiosis. To do this, we conducted a functional rescue experiment. Specifically designed siRNAs were injected into fully grown oocytes to knock down SIRT3 (SIRT3‐KD; Figure 3A,B). Notably, as shown in Figure 3C,D, we found that the spindle defects and chromosome misalignment in SIRT3‐KD oocytes were partially prevented by the overexpression of non‐acetylated GSK3β‐K15R mutant (38.7 ± 4.1% SIRT3‐KD vs 18.4 ± 3.5% SIRT3‐KD + K15R; *P* < .05). In contrast, GSK3β‐K15R did not lower the elevated ROS level in SIRT3‐KD oocytes (22.22 ± 8.23 SIRT3‐KD vs 24.03 ± 9.82 SIRT3‐KD + K15R; *P* > .05; Figure 3E,F). Together, these results indicate that K15 is an important deacetylation site on GSK3β mediating the effects of SIRT3 on meiotic apparatus in oocytes.

### 3.4 Deacetylation‐mimetic mutant GSK3β‐K15R alleviates the meiotic defects in oocytes from diabetic mice

**FIGURE 4:**
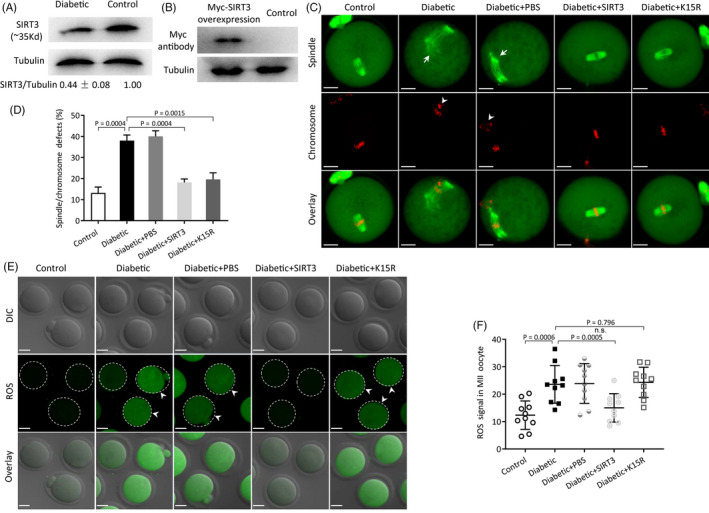
GSK3β‐K15R alleviates meiotic defects in oocytes from diabetic mice. A, Western blot analysis showed the reduced SIRT3 expression in oocytes from diabetic mice compared with controls. Western blot experiments were repeated at least three times, with a representative gel image shown. B, Western blot analysis showed that exogenous SIRT3 protein was efficiently overexpressed. C, Representative images of spindle/chromosomes from control, diabetic, diabetic + PBS, diabetic + SIRT3 and diabetic + K15R oocytes, respectively. Scale bars: 20 μm. D, Percentage of oocyte with abnormal spindle/chromosomes in control, diabetic, diabetic + PBS, diabetic + SIRT3 and diabetic + K15R group. Data are expressed as mean ± SD from three independent experiments in which at least 100 oocytes were examined. E, Representative images of ROS signal (green) in control, diabetic, diabetic + PBS, diabetic + SIRT3 and diabetic + K15R oocytes, respectively. Scale bars: 30 μm. Arrowhead indicates the significantly elevated fluorescence intensity. F, Quantification of ROS fluorescence intensity (n = 10 for each group)

Numerous reports have shown that oocytes from diabetic mice have metabolic dysfunction and meiotic defects,^40^ but the responsible mechanisms remain to be explored. Consistent with previous data, we found that diabetic oocytes display higher frequency of spindle/chromosome defects (38.0 ± 2.7% vs 13.2 ± 2.8% control; *P* < .05; Figure 4C,D) and excessive ROS (23.59 ± 6.87 vs 12.34 ± 5.20 control; *P* < .05; Figure 4E,F) in relative to control cells. On the other hand, we detected a reduction in SIRT3 protein in oocytes from diabetic mice(Figure 4A), and importantly, forced expression of exogenous SIRT3 protein (Figure 4B) significantly ameliorates the meiotic defects (38.0 ± 2.7% diabetic vs 18.2 ± 1.6% diabetic + SIRT3; *P* < .05; Figure 4C,D) and oxidative stress observed (23.59 ± 6.87 diabetic vs 15.01 ± 5.18 diabetic + SIRT3; Figure 4E,F) in diabetic oocytes. Next, we asked whether GSK3β mutant could partly reduce the incidence of meiotic defects in diabetic oocytes. For this purpose, non–acetylation‐mimetic mutant of GSK3β (GSK3β‐K15R) was injected into fully grown oocytes from diabetic mice. Following in vitro maturation, the relevant phenotypes were evaluated. As presented in Figure 4C,D, confocal microscopy revealed that GSK3β‐K15R markedly decreases the percentage of spindle/chromosome defects in diabetic oocytes (38.0 ± 2.7% diabetic vs 19.6 ± 3.1% diabetic + K15R; *P* < .05). In line with the data mentioned above, this constitutively non‐acetylated form of GSK3β was unable to decrease the ROS level in diabetic oocytes (23.59 ± 6.87 diabetic vs 24.33 ± 5.50 diabetic + K15R; *P* > .05 Figure 4E,F). Altogether, these results suggest that SIRT3‐modulated GSK3β‐K15 deacetylation is directly linked to meiotic defects in oocytes from diabetic mice.

## 4 DISCUSSION

GSK3 was linked to glycogen metabolism during insulin signalling in past decades since it was first identified in 1980.^29^, ^41^, ^42^ Recently, increasing evidence demonstrated that GSK3β acts as a signal transduction hub participating in many cellular events, such as apoptosis,^43^ embryonic development,^44^ tumorigenesis^45^ and microtubule dynamics.^46^ Using ATP‐competitive inhibitors, GSK3β has been implicated in spindle assembly and chromosome segregation during mitosis.^31^, ^47^, ^48^ In the present study, we utilized the specifically designed siRNAs to investigate the role of GSK3β in oocyte meiosis. Proper formation of meiotic structure is crucial for maintaining oocyte quality. This study identified GSK3β as a cytoskeletal regulator that is required for this process. Substantial reports have suggested that GSK3β activity is regulated by various mechanisms, such as intracellular localization, binding protein and post‐translational modifications including phosphorylation, ADP‐ribosylation and ubiquitination.^29^, ^49^, ^50^ Most recently, Sundaresan et al^28^ discovered that GSK3β activity is closely associated with its acetylation status controlled by SIRT3. Here, we found that, in normal mouse oocytes, only GSK3β‐K15Q mutant disrupts the maturational progression and assembly of meiotic apparatus (Figure 2), whereas GSK3β‐K15R is able to suppress the abnormal spindle/chromosomes in oocytes depleted of SIRT3 (Figure 3). Our findings support a concept that SIRT3‐controlled GSK3β‐K15 deacetylation plays an important role in the formation of meiotic structure. We cannot rule out that SIRT3 may act on other targets in its function during oocyte maturation. In addition, because of the limited materials and technical reason, we are currently incapable of directly dissecting the relationship between GSK3β acetylation and SIRT3 activity in mouse oocytes. Additional experiments will be required to uncover these details.

Maternal diabetes has been demonstrated to adversely affect preimplantation embryo development and pregnancy outcomes. Emerging evidence has implicated that these effects are associated with compromised oocyte competence.^51^ Specifically, an increased frequency of spindle disorganization and chromosome congress failure was detected in oocytes from diabetic mice.^4^ The molecular mechanism by which maternal diabetes affects meiotic structure in mammalian oocytes has yet to be investigated. Here, we noted that expression of the non–acetylation‐mimetic mutant GSK3β‐K15R effectively lowered the frequency of meiotic defects in diabetic oocytes (Figure 4), which may open a new area for future application of GSK3β deacetylation agonists to treat diabetic patient with reproduction issues. Besides, diabetic oocytes also experience abnormal cellular metabolism and mitochondrial dysfunction.^4^, ^52^ Formation of ROS is a by‐product of oxidative phosphorylation in the mitochondria. It has been widely reported that ROS level was markedly increased in oocytes from diabetic mice when compared to those from control mice.^51^ We have previously revealed a protective mechanism of SIRT3 against oxidative stress in diabetic oocytes via deacetylating SOD at K68.^26^ Interestingly, in the present study, we noticed that ectopic expression of GSK3β‐K15R has little effects on the ROS overproduction in diabetic oocytes (Figure 4). We speculated that SIRT3 plays its protective role through SIRT3‐GSK3β deacetylation pathway and antioxidant system, respectively, in diabetes mice, and ROS appears not be a mediator in this deacetylation pathway. Further studies are imperative to screen candidates that serve as downstream targets of GSK‐3β in SIRT3‐GSK3β deacetylation pathway. Based on these observations, we propose a model where maternal diabetes induces the loss of SIRT3 in oocytes, which, in turn, increases the acetylation of GSK3β‐K15 and impairs its enzymatic activity, consequently resulting in the deficient meiotic apparatus during oocyte maturation. Given the involvements of SIRT3‐GSK3β pathway in diabetic oocytes, prevention of these related deficits transmission may provide therapeutic opportunities for the treatment of reproductive complications and birth defects of diabetic patient.

## CONFLICT OF INTEREST

The authors declare that there is no conflict of interests.

## AUTHOR CONTRIBUTIONS

QW conceived and designed the experiments. YX, YJ, JG, ZH, CL, DW, SZ and LH performed the research and analysed the data. QW and YX interpreted the data and wrote the paper. All authors have read and approved the final manuscript.

## Supporting information

Table S1

## Data Availability

The data that support the findings of this study are available from the corresponding author upon reasonable request.
