# Supplementary material for: Involvement of SIRT3‐GSK3β deacetylation pathway in the effects of maternal diabetes on oocyte meiosis
Source: Cell Prolif. 2020 Oct 26;54(1):e12940. doi: 10.1111/cpr.12940 (PMC7791178; doi:10.1111/cpr.12940)
Supplement: Supplementary file 1 — Table S1 [file CPR-54-e12940-s001.htm]

# Supplemental Table 1

# Primer sequences of gene for cloning

|  |  |
| --- | --- |
| ***Gene*** | ***Primer sequence*** |
| GSK3¦Â | F: 5'-GGGGGCCGGCCGATGTCGGGGCGACCGAGAA-3'  R: 5'-GGGGGCGCGCCTCAGGTGGAGTTGGAAGCTGAT-3' |
| SIRT3 | F: 5'-GGGGGCCGGCCCTGCAGTAGGGTGGTGGTCATG-3'  R: 5'-GGGGGCGCGCCTTATCTGTCCTGTCCATCCATCCATCC-3' |
| K15Q | F: 5'-TGCGGAGAGCTGCCAGCCAGTGCAGCAGC-3'  R: 5'-GCTGCTGCACTGGCTGGCAGCTCTCCGCAA-3' |
| K15R | F: 5'-TTGCGGAGAGCTGCAGGCCAGTGCAGCAGCC-3'  R: 5'-GGCTGCTGCACTGGCCAGCAGCTCTCCGCAA-3' |
| K36Q | F: 5'-GATAAAGATGGCAGCCAGGTAACCACAGTAG-3'  R: 5'-CTACTGTGGTTACCTGGCTGCCATCTTTATC-3' |
| K36R | F: 5'-GATAAAGATGGCAGCAGGGTAACCACAGTAG-3'  R: 5'-CTACTGTGGTTACCCTGCTGCCATCTTTATC-3' |

 

 

# SIRT3 siRNA, GSK3¦Â siRNA and control siRNA sequences

|  |  |
| --- | --- |
| SIRT3 siRNA | F: 5¡¯-GAGUCCUCGAAGGAAAGAUTT-3¡¯ |
|  | R: 5¡¯-AUCUUUCCUUCGAGGACUCTT-3¡¯ |
| GSK3¦Â siRNA | F: 5¡¯-GCGGGACCCAAAUGUCAAATT-3¡¯ |
|  | R: 5¡¯-UUUGACAUUUGGGUCCCGCTT-3¡¯ |
| Negative control sequence | |
|  | F: 5¡¯-UUCUUCGAACGUGUCACGUTT-3¡¯ |
|  | R: 5¡¯-ACGUGACACGUUCGGAGAATT-3¡¯ |

 

 

# Primer sequences of genes for qRT-PCR

|  |  |
| --- | --- |
| ***Gene*** | ***Primer sequence*** |
| SIRT3     GSK3¦Â     GAPDH | F: 5¡¯ -TACAGGCCCAATGTCACTCA-3¡¯  R: 5¡¯ -ACAGACCGTGCATGTAGCTG-3¡¯  F: 5¡¯ -TGGCAGCAAGGTAACCACAG-3¡¯  R: 5¡¯ -CGGTTCTTAAATCGCTTGTCCTG-3¡¯  F: 5¡¯ -CTTTGTCAAGCTCATTTCCTGG-3¡¯  R: 5¡¯ -TCTTGCTCAGTGTCCTTGC-3¡¯ |
